# Short-term zinc supplementation of zinc-deficient seniors counteracts CREMα - mediated IL-2 suppression

**DOI:** 10.1186/s12979-022-00295-8

**Published:** 2022-08-30

**Authors:** Bastian Robinson Baarz, Thea Laurentius, Jana Wolf, Inga Wessels, Leo Cornelius Bollheimer, Lothar Rink

**Affiliations:** 1 Institute of Immunology, Faculty of Medicine, RWTH Aachen University Hospital, Pauwelsstraße 30, 52074 Aachen, Germany; 2 Department of Geriatric Medicine, Faculty of Medicine, RWTH Aachen University Hospital, Morillenhang 27, 52074 Aachen, Germany

**Keywords:** Aging, Cytokines, Interleukin-2, Dietary supplements, Trace element, Zinc

## Abstract

**Background:**

Aging is accompanied by a dramatic decline in the interleukin (IL)-2 production capacity of human immune cells, thus making seniors more susceptible to a variety of age-related diseases. A common cause of impaired cytokine production in advanced age is a deficiency of the essential micronutrient zinc. Nevertheless, the molecular mechanisms underlying a zinc deficiency-induced decrease in IL-2 production have not yet been satisfactorily elucidated. Recent animal and in vitro data suggested that the transcription factor cAMP-responsive element modulator (CREM) $\alpha$ plays a critical role in T cells´ disturbed IL-2 production in suboptimal zinc conditions. However, its role in the human aging process and the possibility of influencing this detrimental process by short-term zinc supplementation have not yet been evaluated.

**Results:**

Comparing peripheral lymphocytes of 23 young and 31 elderly subjects with either high, intermediate, or deficient zinc status, we observed zinc-dependent regulation of the IL-2 production mediated by the transcription factor CREM $\alpha$. For the first time in humans, we report a mutual relationship between low zinc levels, high CREM $\alpha$ expression, subsequent impaired IL-2 production, and vice versa. Remarkably, an average of only 6 days of in vivo zinc supplementation to zinc-deficient seniors was sufficient to rapidly improve zinc status, reverse CREM $\alpha$ overexpression, and counteract subsequent low IL-2 production rates.

**Conclusions:**

Our ex vivo and in vivo data identify zinc deficiency-mediated CREM $\alpha$ overexpression as a key cellular mechanism underlying impaired IL-2 production in the elderly and point toward the use of zinc as a rapidly immune-enhancing add-on nutraceutical in geriatric therapy.

**Graphical abstract:**

During the aging process, there is a progressive decrease in zinc status, which in turn leads to overexpression of the transcription factor CREM$\mathrm{\alpha }$ in peripheral lymphocytes. CREMα is a negative regulator of the IL-2 gene, the overexpression of which dramatically limits adequate IL-2 production. This deleterious mechanism can be counteracted by short-term oral zinc administration, which can adjust IL-2 production in old, zinc-deficient individuals to a level similar to that of young adults.
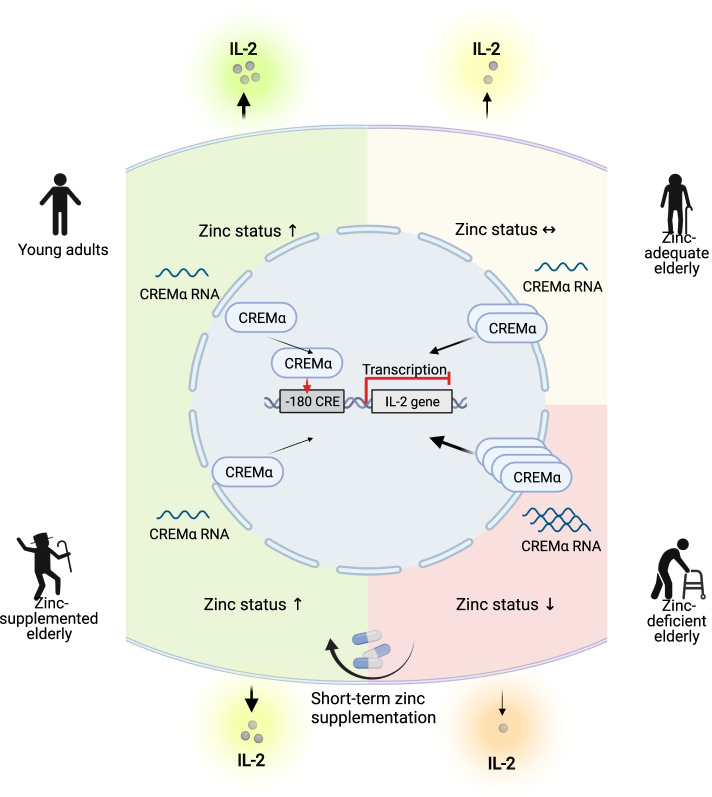

## Background

The human aging process is well described to not spare the immune system [1, 2]. As part of the search for potentially mitigable or even reversible age-related immune impairments, the suboptimal zinc status of old persons came into focus early on [3, 4].

Despite of the lack of a reliable biomarker to accurately assess the zinc status, it is undisputed that zinc status becomes inadequate with advancing age [5, 6]. In terms of the overall population, the prevalence of zinc deficiency (ZD) appeared to be highest in the subgroup of hospitalized elderly, consistent with the poor immune defense of this special cohort [7, 8]. Such an age-related decline in zinc status is mainly due to a mixture of low dietary intake, an age-related detriment in taste, unfavorable subcellular and anatomical changes in the digestive tract, and polypharmacy [9, 10].

Zinc is an essential cofactor for thymulin, the thymic hormone required for T cell differentiation and proliferation, regulation of mature T cell function, and T cell cytokine response to antigenic/mitogenic stimulation [11, 12]. In turn, ZD is causative of decreasing the ability of T cells to produce adequate levels of cytokines in response to stimulation [13, 14]. While some proinflammatory cytokines, such as interleukin 6 (IL-6) are released in greater amounts (inflammaging), the production of other cytokines such as IL-2 decreases sharply in old age as well as in ZD [15–17].

The unbalanced stimulation-induced cytokine production of peripheral lymphocytes subsequently contributes to seniors´ limited vaccination response, increased susceptibility to diseases such as infections and cancer, and to their higher overall mortality [18–20].

Nevertheless, the precise molecular mechanisms that may underlie the impact of either optimized or suboptimal zinc status on late life cytokine regulation remain unthoroughly understood.

In the present report, we first evaluated the existence of a distinctive molecular mechanism of zinc deficiency-triggered IL-2 suppression in human peripheral blood mononuclear cells (PBMC).

In preliminary studies using zinc-deficient human T cell lines and porcine PBMC supplemented with moderate doses of zinc in vitro, our group was the first to demonstrate that the transcription factor CREM $\alpha$ is upregulated in a ZD-dependent manner, thereby actively silencing the IL-2 gene and subsequent IL-2 secretion. The molecular mechanism involves binding of CREM $\alpha$ to the -180 cAMP responsive element (CRE) site within the IL-2 promoter (-164 to -189 bp) upstream of the transcription start, thereby actively inhibiting the initiation of IL-2 gene transcription in a chromatin-remodeling manner [21]. Since the transferability of those in vitro results remained questionable, a major aim of this project was to verify the proposed mechanism for aged and ZD humans. Additionally, we also wanted to check whether it is possible to counteract potential CREM $\alpha$ overexpression in ZD seniors via in vivo short-term zinc supplementation (ZS) and thus increase the IL-2 production to an appropriate level.

To address these questions, we isolated primary human PBMC from subjects of two distinctive cohorts: a healthy, young control cohort, likely to show proper IL-2 production based on a high zinc status, and a frail, elderly cohort, further subdivided into either zinc-adequate (ZA) or ZD subjects at risk of displaying impaired immune response indicated by low IL-2 production.

## Results

### Baseline characteristics of study participants

**Fig. 1:**
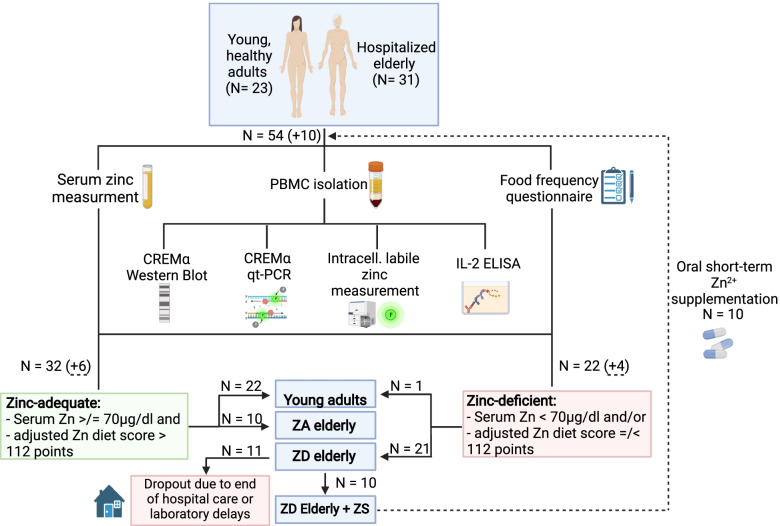
Study design flowchart. We evaluated the effect of different zinc statuses on peripheral blood mononuclear cells (PBMC) CREMα protein as well as RNA expression and subsequent IL-2 production capacity. Overall zinc status was assessed by measuring serum as well as PBMC intracellular labile zinc and applying a zinc-specific food frequency questionnaire to estimate dietary zinc intake. In this study, we compared 23 young, healthy adults to 31 hospitalized geriatric inpatients, further dividing the latter into either zinc-adequate (ZA) or zinc-deficient (ZD). A total of 10 seniors that were previously classified as zinc-deficient by the means of serum zinc or zinc uptake received short-term zinc supplementation (ZS), of whom, after all experiments were repeated, 6 were considered zinc-adequate and 4 were still mildly zinc-deficient (dotted lines)

Between October 2020 and October 2021, a total of 23 young subjects (aged ≤ 31 years) and 31 old subjects (aged ≥ 71 years) participated in the study conducted at RWTH Aachen University Hospital. Table 1 provides details of participants' gender, age, body mass index (BMI), and diet for all study participants, while for obvious reasons the cause for hospitalization, length of hospital stay, major pre-existing conditions, frailty, and medication use are provided only for hospitalized elderly. Interestingly, among the 31 old participants, 21 subjects were classified as ZD by the means of the food frequency questionnaire (FFQ) and/or serum zinc measurements, whereas the only vegan subject belonging to the healthy control was the only one classified as ZD in the young cohort. This corresponds to a ZD prevalence of 68% in the hospitalized elderly and 4% in the young control. 10 ZD elderly received short-term ZS, whereas 11 ZD elderly did not receive short-term ZS because participants' serum zinc measurements were either delayed or their regular hospital care ended before ZS could be started (Fig. 1). After subdividing the 31 aged participants according to their zinc status into ZA, ZD, or ZD and short-term ZS, we could not detect any significant difference in the occurrence of either gender (Chi-square test, ZA vs. ZD: (*p* = 0.53), ZA vs. ZD + ZS: (*p* = 0.65), ZD vs. ZD + ZS: (*p* = 0.92)).

**Table 1:** Characteristics of the study participants

**Characteristics**	Young control *N* = 23	ZA elderly *N* = 10	ZD elderly *N* = 21	ZD elderly + ZS *N* = 10
**Gender**^**1**^**,** n (%)				
male	12 (52)	5 (50)	8 (38)	4 (40)
female	11 (48)	5 (50)	13 (62)	6 (60)
**Age**^**2**^**,** years				
mean ± SD	24.2 ± 3.0^**b**^	84.6 ± 8.1^**a**^	85.7 ± 7.0^**a**^	86.2 ± 5.0^**a**^
median	23	86.5	85	86.5
min, max	20, 31	72, 96	71, 100	80, 94
**BMI**^**3**^**,** kg/m^2^				
mean ± SD	23.5 ± 2.1^**a**^	24.6 ± 4.4^**a**^	25.9 ± 5.8^**a**^	24.2 ± 5.4^**a**^
median	24	24.8	24.7	24.5
min, max	18.4, 26.3	16.2, 33.8	14.9, 41.1	14.9, 35.2
**Diet form,** n (%)				
omnivorous	16 (70)	10 (100)	21 (100)	10 (100)
vegetarian	6 (26)	0 (0)	0 (0)	0 (0)
vegan	1 (4)	0 (0)	0 (0)	0 (0)
**Major hospital admission cause**^**4**^**,** n (%)				
geriatric trauma	-	5 (50)	12 (57)	5 (50)
cardiovascular disease	-	2 (20)	8 (38)	5 (50)
others (metabolic, respiratory, or neurological disease)	-	3 (30)	1 (5)	0 (0)
**Length of hospital stay**^**3**^**,** days				
mean ± SD	-	13.3 ± 6.8^**a**^	25.8 ± 18.7^**a**^	23 ± 10.7^**a**^
median	-	13.5	20	19.5
min, max	-	5, 26	4, 99	4, 44
**Major pre-existing conditions**^**5**^**,** n (%)				
cardiovascular disease	-	10 (100)	21 (100)	10 (100)
metabolic disease	-	9 (90)	13 (62)	8 (80)
kidney disease	-	3 (30)	12 (57)	5 (50)
musculoskeletal disease	-	5 (50)	9 (43)	5 (50)
respiratory disease	-	2 (20)	8 (38)	3 (30)
others (neurological, digestive, urogenital disease)	-	8 (80)	17 (81)	7 (70)
**Hospital Frailty Risk score**^**3**^				
mean ± SD	-	9.0 ± 6.4^**a**^	8.8 ± 6.0^**a**^	7.5 ± 3.8^**a**^
median	-	7.7	8.5	7.2
min, max	-	1.4, 21	0, 25.1	2.9, 14.8
**Medication used at hospital admission**^**5**^**,** n (%)				
proton pump inhibitors	-	7 (70)	18 (86)	8 (80)
diuretics	-	6 (60)	17 (81)	7 (70)
anticoagulants/PAI	-	6 (60)	15 (71)	8 (80)
analgesics	-	4 (40)	15 (71)	7 (70)
ACEi/ARB	-	7 (70)	12 (57)	4 (40)
beta-blockers	-	7 (70)	10 (48)	4 (40)
other antihypertensives	-	1 (10)	9 (43)	2 (20)
statins	-	2 (20)	9 (43)	6 (60)
antidiabetics	-	2 (20)	3 (14)	3 (30)
steroids	-	1 (10)	2 (10)	2 (20)
others	-	5 (50)	14 (67)	6 (60)

*Abbreviations*: *BMI* Body mass index, *ZA* Zinc-adequate, *ZD* Zinc-deficient, *ZS* Zinc supplementation, *PAI* Platelet aggregation inhibitors, *ACEi* Angiotensin-converting enzyme inhibitors, *ARB* Angiotensin-II receptor blockers

^1^ Chi-square test (in this case: all *p* > 0.05, see text for details)

^2^ Kruskal–Wallis test and Dunn´s post-test. Means not sharing any letter (^a^ or ^b^) indicate statistical significance (*p* < 0.05)

^3^ One-way ANOVA and Tukey´s post-test. Means not sharing any letter (^a^ or ^b^) indicate statistical significance (*p* < 0.05)

^4^ under categories; while a hospitalization can, of course, have several reasons, for the sake of clarity we decided to indicate the most predominant one in each case

^5^ under categories

The mean age of all young controls (24.2 ± 3.0 years) was significantly lower than the mean age of all seniors (85.4 ± 7.3 years) (unpaired Student´s t test, (*p* < 0.05)). There were no differences in the mean age of ZA elderly (84.6 ± 8.1 years), ZD elderly (85.7 ± 7.0 years), and ZD elderly + ZS (86.2 ± 5.0 years) (Kruskal–Wallis test and Dunn´s post-test, (*p* = 0.90)).

It is also worth mentioning that all subjects in the old cohort were omnivores, while 6 subjects in the young control group were exclusively vegetarian and one was vegan.

Remarkably, the 10 ZA elderly controls were hospitalized less than half as long as the 11 ZD elderly subjects that did not receive ZS with values of 13.3 days and 28.3 days, respectively (Mann–Whitney-Test, (*p* = 0.0056)). To assess the general physical constitution of the elderly study participants, frailty was calculated according to the Hospital Frailty Risk Score [22]. The scores of ZA 9.0 ± 6.4, ZD 8.8 ± 6.0, and ZD + ZS elderly 7.5 ± 3.8 (mean ± SD), were all in a range considered as an intermediate risk for frailty.

### Zinc status declines with age at the uptake, serum, and intracellular levels

First, we aimed to accurately determine the baseline zinc status of each study participant. On the one hand, we wanted to verify whether the prevalence of ZD in the selected cohort of hospitalized elderly subjects was indeed as high as described in previous studies [23, 24]. On the other hand, the initial determination of the zinc status of each subject allowed us to interpret the data obtained by CREM $\alpha$ and IL-2 measurements and to test them for possible mutual correlations.

**Fig. 2:**
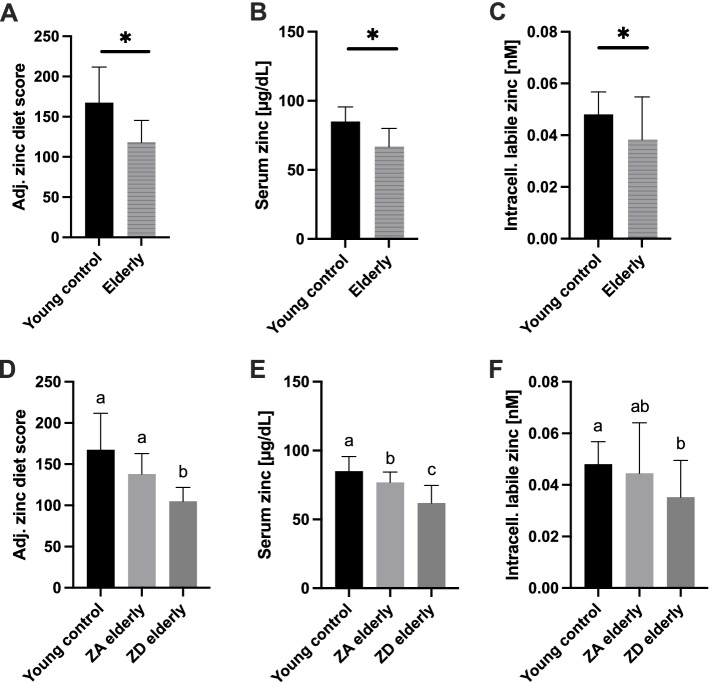
Baseline zinc status of the cohorts. **A-C** depicts the population of all seniors compared to the young control in terms of dietary intake (**A**), serum zinc concentration (**B**), and labile zinc inside peripheral blood mononuclear cells (PBMC) (**C**) After applying predefined cut-off values, the cohort of all geriatric inpatients was further subdivided into either zinc-adequate (ZA) elderly or zinc-deficient (ZD) elderly depending on the zinc status measurement method (**D-F**). Analysis of adjusted zinc diet score and serum zinc of young controls included data of *n* = 23, intracellular labile zinc includes data obtained from *n* = 21 young controls. Regarding the elderly, data from *n* = 31 (**A**, **B**), *n* = 30 (**C**) and depending on whether ZA elderly or ZD elderly are considered, *n* = 10, *n* = 20 (**D** + **F**) and *n* = 9, *n* = 21 (**E**), respectively, were included in the analysis. All data are considered unpaired parameters and presented as the mean + SD. Statistical significance was calculated by Mann–Whitney test in the case of non-normal distribution (**A**), and unpaired t-test in normal distribution (**B**, **C**) (all *(*p* < 0.05)). In **D-F** ordinary one-way ANOVA followed by Tukey´s post-test was used for calculation with means not sharing any letter (a-c) indicating statistical significance (*p* < 0.05)

From the pool of more than 30 proposed biomarkers for determining an individual's zinc status, we selected 3 that we considered sufficiently accurate to approximate subjects’ zinc supply [25]. (i) Dietary zinc uptake was estimated by applying an established FFQ specialized on zinc, calculating an adjusted (adj.; phytate-corrected) zinc diet score [26]. Comparing the adj. zinc diet scores of the young controls to the entire old population net zinc intake was shown to significantly decline (Fig. 2A). Similar results were obtained when analyzing the serum zinc concentration (ii) as the most common marker to determine the human zinc status. The serum zinc was 85 ± 10.6 µg/dL (mean ± SD) in the young group and 67 ± 13.0 µg/dL in the pooled cohort of all elderly subjects (Fig. 2B). In a next step, we checked whether the low serum zinc levels found in the elderly cohort were paralleled by decreased intracellular zinc levels (iii). Therefore, we used the membrane permeable zinc-selective fluorescent probe FluoZin-3 AM, aiming to detect biologically active intracellular zinc [27]. With a mean value of 0.038 ± 0.009 nM, the intracellular zinc levels in PBMC obtained from old subjects was approx. 25% lower than that of PBMC from young subjects (0.048 ± 0.017 nM (both mean ± SD)) (Fig. 2C).

Since one of our goals was to investigate the effects of ZD, we divided the pooled elderly group into either ZA elderly or ZD elderly after applying established cut-off values for the zinc score and serum zinc. The dietary net zinc intake of ZA elderly was significantly higher than that of ZD elderly but still below the zinc score of young participants (*p* = 0.058) (Fig. 2D). Considering serum zinc, the young control subjects reflected the highest mean value of 85 ± 10.6 µg/dL, followed by the ZA seniors, who could barely be classified as ZA with a mean serum zinc concentration of 76.9 ± 7.4 µg/dL, and a serum value of 61.9 ± 12.7 µg/dL in the ZD senior group (Fig. 2E). Thereby the participants can be referred to high (young adults), intermediate (ZA elderly), and low (ZD elderly) in zinc. The free intracellular zinc concentrations in PBMC from ZD elderly donors were significantly lower than in PBMC from young donors, but neither group differed significantly from the elderly ZA group (young control vs. ZA elderly:* p* = 0.78, ZA elderly vs. ZD elderly: *p* = 0.19) (Fig. 2F).

### Uptake, serum, and intracellular zinc determine CREM $\boldsymbol{\alpha }$ protein and RNA expression levels in primary human PBMC

**Fig. 3:**
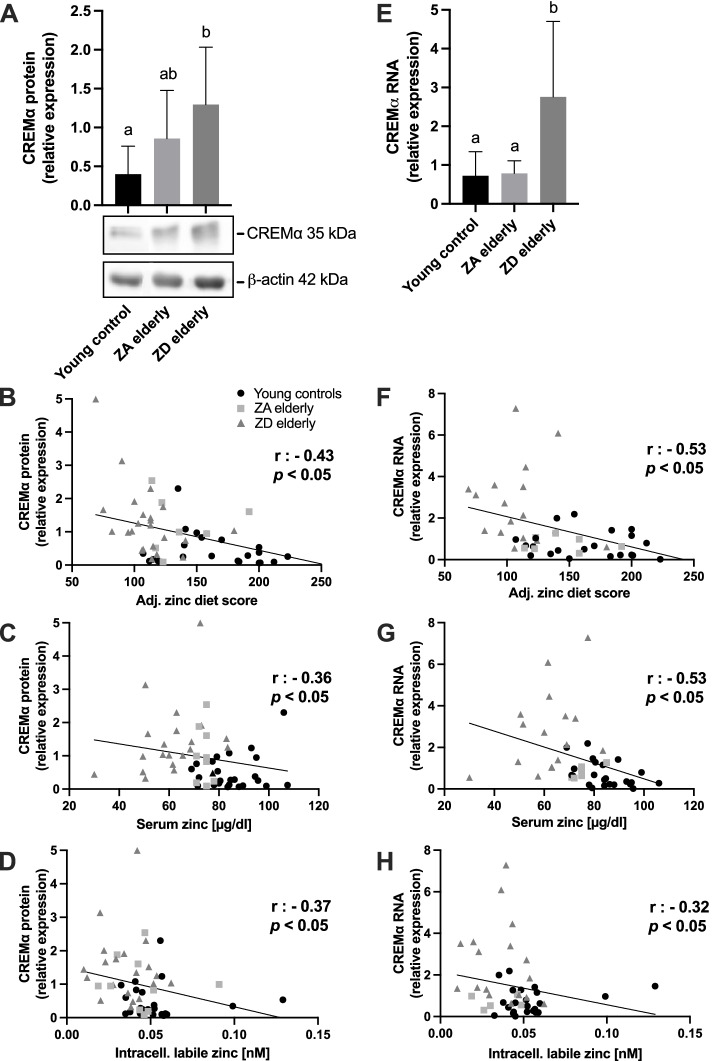
Zinc status determines primary human PBMC CREM $\alpha$ expression. Both basal CREM $\alpha$ protein (**A-D**) and RNA (**E-H**) expression in peripheral blood mononuclear cells (PBMC) depending on zinc status is presented. **A** shows increasing CREM $\alpha$ protein expression between young controls (*n* = 22), ZA elderly patients (*n* = 9) and ZD elderly patients (*n* = 21), as well as a representative Western blot using CREM $\alpha$ and β-actin antibodies. Considering these groups pooled, the adjusted zinc diet score (**B**), serum zinc (both 54 XY pairs) (**C**), and intracellular labile zinc (53 XY pairs) (**D**) correlated negatively with CREM $\alpha$ protein expression. (**E**) Increased basal CREM $\alpha$ RNA expression in elderly ZD patients (*n* = 16) compared to young controls (*n* = 22) and elderly ZA patients (*n* = 8) were detected. CREM $\alpha$ RNA was normalized to the housekeeping gene PBGD. The trend was also confirmed when pooling all groups and plotting the corresponding zinc status indicators against CREM $\alpha$ RNA expression. CREM $\alpha$ RNA expression correlates negatively with adjusted zinc diet score (**F**), serum zinc (both 46 XY pairs) (**G**) and intracellular labile zinc (45 XY pairs) (**H**). Data are shown as means + SD. Differences between the three groups in **A** and **E** were determined by Kruskal–Wallis and Dunn´s post test. Means not sharing any letter (a or b) are significantly different (*p* < 0.05). Correlations were determined by the Spearman rank correlation coefficient (r, *p* < 0.05)

To investigate the impact of differences in zinc status on the expression of the IL-2 gene-regulating transcription factor CREM $\alpha$, we initially compared CREM $\alpha$ protein and RNA levels in primary human PBMC among three groups characterized by either high, intermediate, or low zinc status. In accordance with the age-dependent decrease in zinc status, Fig. 3A shows that basal CREM $\alpha$ protein expression approximately doubles between the young controls and the ZA elderly, whereas it nearly triples compared to the ZD elderly (*p* = 0.24, *p* < 0.05, respectively).

To confirm the hypothesis of strongly zinc-regulated CREM $\alpha$ expression, we additionally plotted the CREM $\alpha$ protein amount of each subject to its corresponding zinc status indicator value. The magnitude of the adj. zinc diet score (Fig. 3B), serum zinc (Fig. 3C), and intracellular labile zinc (Fig. 3D) correlated with CREM $\alpha$ protein levels in a significantly negative manner.

Figure 3 E illustrates the relative CREM $\alpha$ RNA expression for the three groups considered. Zinc-deficient elderly subjects display strongly increased CREM $\alpha$ RNA expression compared with the other two groups assumed to be adequately supplied with zinc. Even group independently, CREM $\alpha$ RNA levels correlate significantly negatively with dietary zinc intake (Fig. 3F), serum zinc concentration (Fig. 3G) and PBMC intracellular labile zinc (Fig. 3H). In summary, our data suggest that the zinc status inversely correlates to CREM $\alpha$ RNA as well as protein levels. Especially given the strong inverse correlations between the zinc supply indicator dietary zinc intake and CREM $\alpha$ RNA levels and between serum zinc and CREM $\alpha$ RNA (both *r* =—0.53, *p* < 0.05), CREM $\alpha$ RNA may represent a new indirect biomarker to assess a person's zinc status

### CREM $\boldsymbol{\alpha }$ expression inversely correlates with IL-2 production by primary human PBMC

**Fig. 4:**
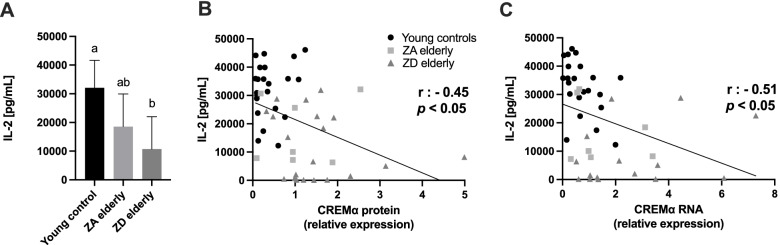
CREM $\alpha$ overexpression suppresses IL-2 production. Depicted is the decreasing interleukin-2 (IL-2) production by stimulated peripheral blood mononuclear cells (PBMC) obtained from the different groups namely young control (*n* = 23), ZA elderly (*n* = 9) and ZD elderly (*n* = 18) (**A**), plotted against basal CREM $\alpha$/β-actin protein expression (49 XY pairs) (**B**) and CREM $\alpha$/PBGD RNA expression (44 XY pairs) (**C**). ZD elderly produced significantly less IL-2 after a 48-h period of mitogenic stimulation with 2.5 µg/mL PHA-L than young controls and less IL-2 than their ZA controls, although the difference was not statistically significant (*p* = 0.29). Both CREM $\alpha$ protein expression (**B**) and CREM $\alpha$ RNA expression (**C**) correlated significantly negatively with the IL-2 production by PBMC. IL-2 levels in the supernatants were quantified by ELISA. Data are shown as means + SD. Significances in (**A**) were calculated using Kruskal–Wallis and Dunn’s post test. Means not sharing any letter (a or b) are significantly different (*p* < 0.05). Correlations were determined by the Spearman rank correlation coefficient (r, *p* < 0.05)

The IL-2 gene is generally regarded as an immune response gene [28]. Undisturbed IL-2 production is therefore considered as an indicator of the integrity of the immune system as a whole and the functionality of T cells in particular. In the subsequent experiment, we first aimed to examine the PBMC of our subjects in terms of their activation-induced ability to produce IL-2. A well-established method for such functional analysis is provided by ex vivo stimulation with the soluble mitogen phytohemagglutinin (PHA)-L [29]. PHA-L is a naturally occurring lectin that is believed to crosslink and activate T cell receptors, thereby inducing the production and secretion of IL-2, among other cytokines [30]. In response to 2 days of PHA-L stimulation, the young subjects produced the greatest amount of IL-2. In the ZA elderly group, IL-2 production halved, albeit non-significantly (*p* = 0.07). The mitogen-induced IL-2 production of the ZD elderly decreased significantly and was only one third of that of the young control (Fig. 4A). This finding is consistent with several other studies that also showed an inverse relationship between age (and zinc status) and IL-2 production [29, 31].

To test if there is a general correlation between CREM $\alpha$ RNA or protein expression with the IL-2 expression, we plotted every study participant´s CREM $\alpha$ expression against its corresponding stimulation-induced IL-2 production. The direct comparison of these parameters clearly confirms a mutual interaction between the transcription factor and the cytokine (Fig. 4B + C). Higher CREM $\alpha$ protein levels were associated with lower IL-2 amounts in the supernatants, indicated by a Spearman correlation coefficient of *r* =—0.45 (*p* < 0.05). The results were even more significant when comparing CREM $\alpha$ RNA levels to the IL-2 amount produced (*r* =—0.51, *p* < 0.05). In accordance with our hypothesis, these findings strongly indicate the suppressive role of CREM $\alpha$ regarding the IL-2 production capacity.

### Short-term zinc supplementation of zinc-deficient elderly individuals improves zinc status and restores IL-2 production via CREM $\boldsymbol{\alpha }$ downregulation

Having shown that CREM $\alpha$ is a highly zinc-dependent transcription factor, which in turn suppresses IL-2 production in a concentration-dependent manner, the question arose whether this detrimental mechanism can be counteracted by improving the zinc status in vivo.

Since a diagnosed ZD is a mandatory indication for ZS, rapid compensation of suboptimal zinc status should be sought immediately. For patient´s benefit, a set-up of a group of zinc-deficient, but non-zinc-receiving seniors was therefore excluded by ethical means.

Nevertheless, elderly with pre-existing ZD are most likely affected by overexpression of CREM $\alpha$ and low IL-2 production and may therefore benefit the most from additional ZS. ZD and subsequent oral short-term ZS were indicated by an adj. zinc-diet score below 113 points and/or by a serum zinc concentration below 70 µg/dL [26, 32]. From the 21 elderly subjects that met the criteria, 10 were successfully supplemented with zinc. The mean duration of zinc supplementation was 5.7 ± 2.8 days. Although difficult to monitor, no side-effects attributable to zinc administration were identified during this time. Likewise, not a single subject discontinued the zinc supplementation. Due to laboratory delays and end of regular hospital care, supplementation could unfortunately not be promptly realized in 11 subjects.

**Fig. 5:**
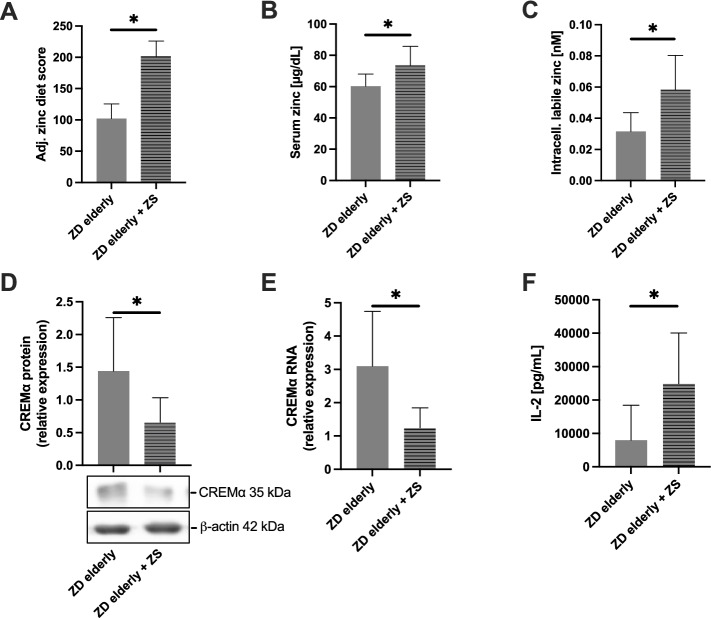
Differential effects of short-term zinc supplementation of zinc-deficient elderly subjects. **A** Significant increase in dietary zinc intake after short-term zinc supplementation (ZS) of zinc-deficient (ZD) seniors as measured by a zinc-specific food-frequency questionnaire calculating an adjusted zinc diet score. A short-term ZS leads to a significant increase in serum zinc concentration (**B**) and freely available zinc (**C**) in peripheral blood mononuclear cells (PBMC) (all *n* = 10). Short-term ZS significantly decreased CREM $\alpha$ protein (**D**) and RNA (**E**) expression in resting PBMC (both *n* = 9). One representative Western blot is shown in (**D**). Short-term ZS significantly increased IL-2 production (**F**) by PBMC after a 48-h period of stimulation with 2.5 µg/mL PHA-L (*n* = 8). IL-2 levels in the supernatants were quantified by ELISA. The figures contain paired parameters only, i.e. individuals who were primarily zinc deficient but completed short-term ZS. Data are shown as means + SD. Normally distributed data in **A**, **B**, **D**, and **E** were tested for significance using paired Student's t test (*(*p* < 0.05)); in the case of **C** and **F**, two-tailed Wilcoxon test was applied due to non-normal distribution (*(*p* < 0.05))

Short-term ZS markedly improved the zinc status of elderly people with pre-existing ZD at all levels considered. While short-term zinc supply almost doubled the adj. zinc diet score (Fig. 5A), serum zinc increased less but still significantly from 60.3 ± 7.8 µg/dL to 73.5 ± 12.3 µg/dL (Fig. 5B) and thus into a range that can be considered ZA. Consistent with the zinc diet score, intracellular freely available zinc also doubled after transient ZS (Fig. 5C).

After demonstrating that daily ZS for approximately 6 days is sufficient to compensate for a suboptimal zinc status, we wanted to analyze whether such an improvement is also sufficient to suppress excessive CREM $\alpha$ production in resting PBMC. This was confirmed at both the transcriptional and protein levels (Fig. 5D + E). Following ZS, CREM $\alpha$ protein and RNA decreased to approximately one-third of baseline.

Finally, we wanted to test whether zinc-induced suppression of CREM $\alpha$ overexpression also leads to a normalization of IL-2 production capacity. In line with our hypothesis, IL-2 concentrations were approximately threefold increased in PBMC supernatants following in vivo ZS (Fig. 5F). This finding further emphasizes the mutual interaction between zinc levels, CREM $\alpha$ expression, and subsequent IL-2 production.

## Discussion

The frequent presence of suboptimal zinc status associated with low IL-2 production capacity of peripheral immune cells has been shown to predispose the elderly to various age-related diseases and contribute to their increased overall mortality [33, 34]. Hence, a thorough understanding of the molecular mechanisms underlying the zinc deficiency-induced IL-2 production impairment may help unravel important immune aging principles and develop potential health-promoting strategies.

In the work presented herein, we observed impaired IL-2 production in the elderly caused by zinc deficiency-dependent CREM $\alpha$ overexpression. Although CREM $\alpha$-mediated IL-2 gene silencing has been described before in other contexts [35] to the best of our knowledge, this is the first description of (i) the existence of zinc deficiency-induced CREM $\alpha$-tiggered IL-2 suppression in (elderly) humans and (ii) zinc posing a potential treatment of suppressed IL-2 production in the elderly via rapid reversibility of CREM $\alpha$ overexpression through transient in vivo supplementation.

The first challenge of this study was to accurately screen each subject in terms of their baseline zinc status. As summarized in detail by Lowe et al., the list of zinc biomarkers includes more than 30 different candidates that vary widely in their accuracy [25]. For this reason, we inferred the baseline zinc status of our study participants assessing not only one but three indicators, all of which aimed to measure different zinc pools.

We calculated a ZD prevalence of 68% among the elderly subjects which even slightly exceeded the values described in other studies [24, 36]. This could be because zinc status is described to decrease in an age-dependent manner, and the average age of our elderly subjects of 85.4 years was higher than that in most comparable studies [32]. Among the young controls, a single subject was classified as mildly ZD (serum zinc: 69 µg/dL), which is unusual but may reflect the low zinc intake from its vegan diet, as this diet form was recently shown to predispose to micronutrient deficiencies [37]. We intentionally chose not to exclude this subject from the dataset, as this would not be consistent with our predefined exclusion criteria but emphasizing that exclusion would certainly have resulted in even stronger significances between the young and the older subjects.

Remarkably, concerning the length of hospital stay, we found that the ZD seniors without ZS were hospitalized significantly longer as their ZA counterparts (mean ZA: 13.3 days, mean ZD w/o ZS: 28.3 days). Considering the well described role of zinc in proper immunity it is not surprising that subjects with pre-existing ZD face a longer hospitalization period than others with adequate zinc status. This is supported by recent data demonstrating that old COVID-19 patients with suboptimal zinc status are hospitalized significantly longer than controls with improved zinc status [38, 39].

We next aimed to evaluate how the current zinc status of each subject affected their basal CREM $\alpha$ expression. All zinc parameters correlated in a significant negative manner with CREM $\alpha$ protein and RNA expression, thereby confirming the reciprocal trend between overall zinc status and CREM $\alpha$ expression, which was first proposed by our laboratory [21]. Given this ZD-induced CREM $\alpha$ overexpression, we subsequently wanted to examine whether the elderly suffered from reduced activation-induced IL-2 production as a result. Consistent with previous findings we observed lower IL-2 production in the elderly, which was exacerbated by pre-existing ZD [29, 31]. Many groups have aimed to mitigate or even reverse such restriction of IL-2 production. Regarding nutrient-based interventions, there are reports that vitamin E supply of aged mice and humans improves IL-2 production capacity [40, 41]. However, this effect was at least in parts considered to be a side effect of a reduction in macrophage-produced T cell suppressive prostaglandin (PG) E2 [42]. The initial description of zinc having a beneficial effect on IL-2 gene expression in human PBMC, dates to in vitro work by Prasad and coworkers [29]. In contrast, there have also been some rare descriptions of zinc suppressing IL-2 production by T cells [43]. This supposed contradiction is most likely due to the differences in experimental protocols such as amounts of zinc used in those studies. Indeed, supraphysiological amounts of zinc were shown to promote regulatory T cell (Treg) induction, which in turn can suppress the T cell response due to IL-2 scavenging via soluble and membrane bound CD25 [44, 45].

Because comparability is an important feature of nutrient supplementation studies, we decided to administer a zinc dose (and formula) that has been used in similar studies and is also in line with the U.S. National Institute of Health's (NIH) recommended daily allowance (RDA) for zinc [46]. ZS was conducted with 10 mg of zinc aspartate daily, an amount that is considered moderate and, most importantly, not described to suppress proper T cell function [14, 47]. Since we wanted to gain new insights into the question of how quickly the zinc status improves in vivo we opted for a shorter period of ZS than in common ZS studies, in which zinc was usually given for several weeks or months [32]. The literature on the topic of short-term zinc supplementation is inhomogeneous. Most studies on this have been conducted in neonates, while studies in seniors are scarce. Even the term itself is inconsistent, leading short-term ZS to vary from a few days to several weeks [48, 49]. We showed, that an average ZS of 5.7 days seems to be sufficient to compensate for suboptimal zinc status. This was reflected by an 20% increase in serum zinc (73.5 ± 12.3 µg/dL, by definition ZA) and an even greater 81% increase of labile intracellular zinc. The fact that suboptimal zinc status can be corrected in such a short period of time may be of great importance for medical care. However, an earlier study carried out with 19 elderly people using the same zinc formula and dose for a mean period of 48 days, found an increase of only 11% and 61% for serum zinc and intracellular free zinc, respectively [47]. Therefore, it may be assumed that the “driving force” of aging at least somewhat counteracts the initial improvement in zinc status in the longer term. This kinetics should be given attention in future studies. At this point, the question may arise whether it is more advisable to supplement with a lower dose of zinc for a longer period or with a higher zinc dose for a shorter period. Based on our data, this question cannot be answered. Although one should always consider the individual case, it can be deduced from current recommendations and risk assessments of the NIH, European Food Safety Authority (EFSA), and other health authorities that it seems more beneficial for health to constantly administer zinc in a low dose [46]. Weißenborn et al. calculated maximum levels for minerals in food supplements, which could be supplied without the risk of adverse effects in addition the regular diet [50]. According to this report, 6.5 mg of zinc per day in addition to regular diet, containing the recommended daily amount of zinc, should be harmless even for individuals with an adequate zinc status. On the other hand, the NOAEL (no observed adverse effect level) for zinc is 0.43 mg/kg body weight, resulting in 25.8 mg per day for an adult of just 60 kg body weight [51]. Therefore, long-term, low dose zinc supplementation should be better for health promotion than short-term, high dose supplementation. Furthermore, we can conclude that early screening for suboptimal zinc status on hospital admission, e.g. by means of a clinically established zinc-specific FFQ, appears to be useful, easy to perform, cost effective and potentially even of higher diagnostic value than serum zinc measurements in non-specialized routine laboratories as shown by Trame and coworkers [26]. Our results suggest that identifying those individuals who might benefit from immediate zinc supply is of clinical importance.

Following in vivo ZS, we observed a significant decrease in CREM $\alpha$ expression paralleled by an approximately threefold increase in IL-2 production. Considering the preceding study demonstrating this mechanism in zinc-deficient and in vitro zinc-supplemented T-cell lines and PBMC, we strongly attribute this zinc effect to the abolished CREM $\alpha$-mediated inhibition of IL-2 gene transcription [21]. The finding of elevated IL-2 levels following zinc supplementation fits preliminary results of a study conducted by Prasad et al. in which 6 initially ZD seniors received a zinc dose equivalent to our study for a period of 6 months. While IL-2 mRNA levels of ZD PBMC were significantly decreased at baseline (*p* = 0.0001), they experienced a significant increase (*p* < 0.05) after ZS compared with a group of 12 ZA, age-matched controls [19]. Together, these findings strongly suggest that zinc status correction may contribute to a substantial improvement of the diminished IL-2 production capacity at old age.

Given these results, future studies conducting short-term ZS may aim to include larger participants numbers and recruit different cohorts and might also evaluate other surrogate parameters for improved T cell response. For instance, in order to exclude any effect of hospitalization it would be interesting to investigate whether the same mechanisms also apply to non-hospitalized elderly. And to further elucidate any specific age-related effect it would be also interesting to pursue supplemention of young hospitalized patients with ZD. However, also in non-hospitalized elderly it would be against good clinical practice to withhold supplemention despite the diagnostic knowledge about ZD. Therefore, a large cohort of elderly ought to be investigated with random zinc or placebo supplementation, where the zinc status at beginning will would have to be unblinded only in hindsight at the end of the supplementation. Due to new regulatory publications, suggesting no more than 6.5 mg of zinc as a dietary supplement to regular diet, it may be critical to obtain approval for such a study in Germany [50]. Although zinc deficiency screening in young, hospitalized patients should be possible in principle, it becomes much more difficult to include a critical number of patients in this age group. This is because (i) the number of young ZD patients is half that of the elderly [52], (ii) the number of patients between 18 and 60 years of age is only a quarter of those over 65 years of age [53], and (iii) length of hospitalization increases with age while most young patients stay in hospital for less than 6 days [54]. Therefore, the number of young patients to be screened must be around 16 times higher than in geriatric medicine.

Future studies may include other known risk groups for ZD and examine them for similar effects. Unfortunately, the small number of subjects on vegan or vegetarian diets included in our study is not sufficient for a powerful subgroup analysis. Furthermore, it should be sought to investigate whether a rapidly improved zinc status is also sufficient to improve T cell proliferation which is often reduced in old age and therefore regarded as another characteristic of immunosenescence.

Despite working in all conscience, our study still has some limitations. As a transcription factor, CREM $\mathrm{\alpha }$'s main area of activity is expected to be in the cell nucleus. Nevertheless, we did not separately assess the nuclear localization of CREM $\mathrm{\alpha }$, which is necessary for transcriptional activity. However, we are certain that such observed CREMα overexpression is also reflected at the nucleus level, as has been demonstrated previously [55].

We are also aware, that it cannot fully be excluded that the study enrollment immediately after hospitalization may have had a confounding effect on some zinc and immune measures in the seniors. However, to avoid this best as possible, we did not include subjects in the study who suffered from common conditions known to affect zinc homeostasis, the immune system, and their measurable parameters. In addition, we were dependent on hospitalized seniors, because, as mentioned earlier, they represent one of the few and easily accessible in vivo collectives for ZD.

Moreover, it unfortunately was not possible to establish a placebo-receiving control group of ZD seniors. As mentioned above, a diagnosed ZD is an obligatory indication for ZS. Leaving such a condition untreated would therefore be negligent and unethical. Although we could not conclusively exclude confounding factors by the selection criteria, we still consider our study design suitable to claim that the measured effects regarding CREMα and IL-2 are primarily due to the observed improvement in zinc status. We believe so for several reasons: On the one hand, our results are fully in line with well-controlled observations in previous in vitro studies [21]. On the other hand, previous placebo-controlled studies including large numbers of subjects with similar baseline characteristics have already shown that zinc status can be improved by moderate zinc supplementation in a dose-dependent manner [19, 56]. Thus, this life-like study approach may even be of clinical importance, as it has already been shown that ZS improves immune status in healthy elderly [47], and, in addition to current knowledge, we hereby provide evidence that the effect appears to be much greater in hospitalized elderly with ZD, since their impairment of the immune response is stronger than in their ZA counterparts. Against this background, and especially considering the large interindividual heterogeneity in the fourth stage of life, we therefore consider this approach reasonable.

## Conclusion

We demonstrated that an age-dependent decline in zinc status is directly associated with increasing CREMα concentrations in human PBMC. As a negative regulator of the IL-2 gene, CREMα overexpression contributes to PBMC hyporesponsiveness, as indicated by lower IL-2 production upon mitogenic stimulation, and therefore may restrict a proper immune response. We show that this condition can be reversed through short-term ZS. Although transient ZS to ZD seniors did not increase IL-2 production to levels completely reaching those of young, healthy subjects, our data indeed indicate that improved zinc status allows aged PBMC to partly regain their function, as evidenced by markedly heightened IL-2 levels. The ZS duration is particularly worth highlighting because, to the best of our knowledge, previous comparable in vivo studies have mainly focused on the effects of continuous long-term zinc administration, but we, in turn, have shown that short ZS may be sufficient to rapidly support proper functioning of peripheral immune cells. Such nutritional interventions are emerging because they not only offer new prophylactic and therapeutic strategies for age-related diseases but are also readily available, low in cost, and with few side effects.

## Methods

### Subject recruitment and study design

This study was conducted from October 2020 until October 2021. The protocol design was reviewed and accepted by the institutional ethics committee of the RWTH Aachen medical faculty (young controls: EK023/05; elderly subjects: EK206/09). The research conforms to the principles of the Declaration of Helsinki. All subjects provided written informed consent before sample collection. The study enrolled young subjects aged 20 to 31 years who were recruited through a call from the Medical Faculty of RWTH Aachen University. Elderly subjects were asked to participate after admission to the geriatric department of the RWTH Aachen University Hospital. Participants consuming omnivorous, vegetarian, or vegan diets were all included in the study. The exclusion criteria for all participants were 1) severe infection with the need for antimicrobial therapy 2) autoimmune diseases with the need for immunosuppressive medication and 3) diagnostically confirmed progressive neoplasia or malignancy of any kind.

The control group consisted of 23 healthy young adults, which were compared to 31 hospitalized elderly individuals. The experimental procedure of the study is shown in Fig. 1. In accordance with comparable studies zinc deficiency was indicated by a serum zinc concentration below 70 µg/dL and/or an adjusted zinc diet score below 113 points [26, 34]. After detecting zinc deficiency, elderly subjects were directly supplemented with 10 mg of zinc aspartate (Köhler Pharma, Albach-Hähnlein, Germany) daily as clinically indicated. The same experiments were performed before and after supplementation.

### Blood collection and isolation of peripheral blood mononuclear cells (PBMC)

After overnight fasting, 15 mL of blood was collected in 7.5 mL lithium-heparin monovettes (Sarstedt, Nürnbrecht, Germany) by venipuncture. Isolation of PBMC using Biocoll Separating Solution 1.077 g/mL (Biochrom, Berlin, Germany) was performed as described elsewhere [27]. Cells were adjusted to a final concentration of 1 × 10^6^ cells/mL in culture medium. The culture medium consisted of RPMI 1640 containing 10% fetal calf serum (FCS, Bio&Sell, Feucht, Germany), 2 mM L-glutamine, 100 U/mL potassium penicillin and 100 U/mL streptomycin sulfate (all Sigma-Aldrich, Steinheim, Germany). Incubation steps were carried out in a humidified 5% CO_2_ atmosphere at 37 °C.

### Assessment and calculation of study participants baseline characteristics

Gender, age, BMI, and diet of study participants were recorded directly at study enrollment. Data regarding the reason for hospital admission, length of hospitalization, major comorbidities, and pre-existing medication were retrospectively analyzed and grouped into categories by two experienced clinicians. The day prior to blood collection dietary zinc uptake was assessed by a nutritional study nurse using an established 18-item food frequency questionnaire (FFQ) specialized on zinc [26]. According to the questionnaire’s instructions, a phytate-corrected zinc diet score was calculated. Because phytates are zinc chelators that decrease intestinal zinc absorption despite adequate amounts of zinc contained in the diet, we decided to calculate this adj. zinc diet score to estimate net zinc uptake. Food frequency, serving types, and quantity were inquired. Additionally, the intake of supplementary zinc products was evaluated. According to the information provided in the FFQ, an adj. zinc diet score of less than 113 points was considered ZD [26].

Frailty is a major geriatric syndrome understood as the age-related decline in function and integrity of the organism. To determine the degree of frailty we decided to calculate it using the HFRS well established in the acute-care setting of seniors [22]. Frailty quantification was done by a study nurse according to the criteria included in the primary publication by Gilbert and coworkers. The thresholds for classifying patients as low frailty risk, moderate frailty risk, or high frailty risk are scores of < 5, 5–15, and > 15, respectively.

### Serum zinc measurement

Serum samples were collected in 2.7 mL serum monovettes suitable for zinc measurement (Sarstedt, Nürnbrecht, Germany) and were allowed to clot for at least 30 min at room temperature. Subsequently, samples were centrifuged for 10 min at 1841 × g. Prior to serum zinc measurement the serum was diluted 1:5 in deionized water and stored in sterile Eppendorf tubes at – 20 °C. The final serum zinc concentration was determined via flame atomic absorption spectrometry by an AAnalyst 800 (Perkin-Elmer, Waltham, United States) as previously described [57]. In accordance with comparable studies serum zinc concentrations below 70 µg/dL were considered zinc-deficient [24, 34].

### Flow cytometric measurement of labile intracellular zinc

Following PBMC isolation, 1 × 10^6^ cells per sample were centrifuged and resuspended in 0.5 mL measurement buffer containing 1 μM zinc selective probe FluoZin-3 AM (Invitrogen, Darmstadt, Germany) as previously described [27]. Afterwards, the samples were incubated for an additional 15 min at 37 °C with either 50 μM of zinc chelator N,N,N,N-tetrakis (2-pyridylmethyl)-ethylenediamine (TPEN) to obtain minimal fluorescence (F_min_), with 100 µM zinc sulfate (ZnSO_4_) in combination with 5 µM sodium pyrithione ionophore (all Sigma-Aldrich, Germany) to obtain maximal fluorescence (F_max_), or were left untreated (F). Subsequent flow cytometry measurements were performed using a FACSCalibur (BD Bioscience, Heidelberg, Germany) and analyzed with FlowJo (TreeStar). Only structurally normal cells were selected for analyses and all samples were gated equally. Calculation of intracellular labile zinc was performed by applying the equation [Zn^2+^] = K_D_ $\times \frac{\mathrm{F}-{\mathrm{F}}_{\mathrm{min}}}{{\mathrm{F}}_{\mathrm{max}}-\mathrm{F}}$, using a dissociation constant K_D_ of 8.9 nM for the Zn^2+^/FluoZin-3 AM complex.

### IL-2 quantification

After PBMC isolation, 1 × 10^6^ cells were stimulated for 48 h with 2.5 μg/mL PHA-L (Pan-Biotech, Aidenbach, Germany) at 37 °C. Supernatants for IL-2 determination were harvested from 1 × 10^6^ cells/mL. Subsequently, supernatants were stored at -20 °C. After sample dilution, IL-2 protein concentrations in supernatants of PBMC were determined by OptEIA ELISA (BD, Heidelberg, Germany) according to the manufacturer’s instructions with a detection limit of 7.8 pg/mL. The intra- and interassay coefficients of variability were calculated to be 4.5% and 8.8%, respectively. Each sample was investigated in duplicate. The absorption was measured using a Spark wellplate reader (Tecan, Crailsheim, Germany).

### Western blotting

After isolation of PBMC, 2 × 10^6^ cells per sample were prepared for protein detection and quantification by Western blot as previously described [21]. One colored prestained standard (New England BioLabs, Frankfurt a.M., Germany) for weight determination was applied in a pocket of each 10% polyacrylamide gel. Primary antibodies against CREM C-2 (Santa Cruz Biotechnology, Heidelberg, Germany) and the housekeeping gene β-actin (Cell Signaling Technology, Frankfurt a.M., Germany) were diluted 1:500 and 1:1000 respectively in tris buffered saline (TBS: 20 mM Tris–HCl, 136 mM NaCl (Applichem, Darmstadt, Germany)), supplemented with 0.1% Tween 20 (Sigma-Aldrich) containing 5% bovine serum albumin (BSA, AppliChem). Membranes were incubated with the primary antibodies overnight at 4 °C. The second day membranes were washed three times with TBS-T, before incubation in secondary antibody. Each secondary antibody, an horseradish peroxidase (HRP)-coupled anti-mouse IgG for CREM C-2 and an HRP-coupled anti-rabbit IgG for β-actin (both Cell Signaling Technology, Frankfurt a.M., Germany), was diluted 1:2000 in TBS-T containing 5% powdered milk and membranes were incubated for 3 h at room temperature. LumiGlo reagent (Cell Signaling Technology, Frankfurt a. M., Germany) was used for visualization of the protein bands. Luminescence was analyzed by using an LAS 3000 (Fujifilm Lifescience, Düsseldorf, Germany) and quantified with ImageJ (NIH). To provide comparability between the different blots, the relative protein expression of each sample was normalized to a corresponding loading control standard administered at every gel.

### Quantitative Real-Time Polymerase Chain Reaction (qRT-PCR)

Following PBMC isolation, 1 × 10^6^ cells of each subject were centrifuged at 300 × g for 5 min. The cell pellet was lysed in 1 mL TRIzol Reagent (Ambion/Life Technologies, Darmstadt, Germany) and stored at -80 °C. mRNA was isolated according to the manufacturer’s instructions and measured with a Nanodrop 2000 (Fisher Scientific, Schwerte, Germany). Transcription into cDNA was performed with the qScript cDNA Synthesis Kit (Quantabio, Darmstadt, Germany) following the manufacturer´s instructions.

Quantitative real-time PCR was performed using fluorescent SYBR green on a QuantStudio3 Real-Time PCR System (Applied Biosystems, Darmstadt, Germany). The following primers were used: CREMα (forward primer: TCG CGA ACT TGG GAC GA, reverse primer: GCT GGG GTG TTG AAG GT), and porphobilinogen deaminase (PBGD) (forward primer: ACG ATC CCG AGA CTC TGC TTC, reverse primer: GCA CGG CTA CTG GCA CAC T) [21]. Reaction conditions were initial 95 °C for 10 min, followed by 40 cycles of 95 °C for 15 s and 60 °C for 60 s. Each sample was analyzed in duplicate. Aqua ad iniectabilia (Braun, Melsungen, Germany) in the equivalent volume to DNA functioned as a no template control. For quality control, every PCR was completed by a melt curve stage (95 °C 15 s, 60 °C 1 min, 95 °C 15 s). With the use of the $\Delta \Delta$ C_T_ method, CREMαexpression was first normalized to the expression of the housekeeping gene PBGD and then normalized to a standard sample administered at every PCR plate.

### Statistics

Statistical analysis was performed using GraphPad Prism (version 9.1). All data were analyzed for outliers, which were removed accordingly as well as tested for normality with the Shapiro–Wilk test. The tests used for significance testing are indicated in the corresponding text passages or figure legends.

The three groups (young control, ZA elderly and ZD elderly) were considered unpaired parameters. Data comparing ZD elderly subjects to ZD elderly + ZS subjects were considered paired. Correlations were determined by calculating the Spearman correlation coefficient r. In all data sets, *p* values < 0.05 were considered statistically significant.

## Data Availability

The datasets generated and/or analyzed during the current study are not publicly available but are available from the corresponding author on reasonable request.
